# Fosfomycin: Uses and potentialities in veterinary medicine

**Published:** 2014-03-16

**Authors:** D.S. Pérez, M.O. Tapia, A.L. Soraci

**Affiliations:** 1 *Laboratorio de Toxicología, Centro de Investigación Veterinaria de Tandil, Departamento de Fisiopatología, Facultad de Ciencias Veterinarias, Universidad Nacional del Centro de la Provincia de Buenos Aires, Tandil, Buenos Aires, Argentina*; 2 *Consejo Nacional de Investigaciones Científicas y Técnicas (CONICET), Buenos Aires, Argentina*

**Keywords:** Antibiotic, Clinical uses, Fosfomycin, Pharmacodynamics, Pharmacokinetics

## Abstract

Fosfomycin (FOS) is a natural bactericidal broad-spectrum antibiotic which acts on proliferating bacteria by inhibiting cell wall and early murein/peptidoglycan synthesis. Bactericidal activity is evident against Gram positive and Gram negative bacteria and can also act synergistically with other antibiotics. Bacterial resistance to FOS may be natural or acquired. Other properties of this drug include inhibition of bacterial adhesion to epithelial cells, exopolysaccharide biofilm penetration, immunomodulatory effect, phagocytosis promotion and protection against the nephrotoxicity caused by other drugs. FOS has chemical characteristics not typically observed in organic phosphoric compounds and its molecular weight is almost the lowest of all the antimicrobials. It tends to form salts easily due to its acidic nature (disodium salt, for intravenous (IV), intramuscular (IM) and subcutaneous (SC) administration; calcium and trometamol salt: for oral (PO) administration). FOS has a very low protein binding (<0.5%) which, along with its low molecular weight and water solubility, contributes to its good diffusion into fluids (cerebrospinal fluid, aqueous and vitreous humor, interstitial fluid) and tissues (placenta, bone, muscle, liver, kidney and skin/fat). In all species, important differences in the bioavailability have been found after administration in relation to the various derivatives of FOS salts. Pharmacokinetic profiles have been described in humans, chickens, rabbits, cows, dogs, horses and weaning piglets. The low toxicity and potential efficacy of FOS are the main factors that contribute to its use in humans and animals. Thus, it has been used to treat a broad variety of bacterial infections in humans, such as localized peritonitis, brain abscesses, severe soft tissue infections, cystitis and other conditions. In veterinary medicine, FOS is used to treat infectious diseases of broiler chickens and pigs. In broilers, it is administered for the treatment of *E. coli* and *Salmonella spp*. infections. In piglets, the drug is prescribed to treat a wide variety of bacterial infections. FOS penetration is demonstrated in phagocytic, respiratory (HEP-2) and intestinal (IPEC-J2) cells. Although not widely used in animals, the drug has shown good results in human medicine. The potentialities of FOS suggest that this drug is a promising candidate for the treatment of infections in veterinary medicine. For these reasons, the aim of this work is to provide animal health practitioners with information on a drug that is not extensively recognized.

## Introduction

Fosfomycin (FOS) (cis-1,2-epoxyphosphonic acid), initially known as ‘phosphonomycin’ (Hendlin *et al.*, 1969), is a natural bactericidal broad-spectrum antibiotic that is not structurally related to other classes of antimicrobial agents (Escolar Jurado *et al.*, 1998; Popovic *et al.*, 2010).

It was isolated in 1966 from a *Streptomyces fradiae* strain, obtained from a soil sample, and later, from *Streptomyces viridochromogenes*, *Streptomyces wedmorensis* (Hendlin *et al.*, 1969; Grassi, 1990), *Pseudomona viridiflava* and *Penicillum* strains (Hidaka *et al.*, 1992; Hidaka *et al.*, 1995; Shi *et al.*, 2001). Currently, it is exclusively produced by chemical synthesis (Gobernado, 2003).

FOS is a Spanish antibiotic, undervalued in the English medical literature and not regularly used in its country of origin (Vargas *et al.*, 1987; Gudiol, 2007). FOS is being used in veterinary medicine for over 40 years. However, it is usually considered a second line antibiotic (Vargas *et al.*, 1987), mainly due to the lack of knowledge among veterinary professionals. This unrecognition of the drug reflects the fact that most of the studies are performed in humans and they are scarce and only recently applied to domestic animal medicine. Nevertheless, FOS is a good antibiotic, with a fast effect, good tolerance (Ilender, 1998) and physicochemical and pharmacokinetic characteristics that allow its enteral and parenteral administration (Dámaso *et al.*, 1990; Mensa *et al.*, 1994).

FOS has a very low protein binding (<0.5%). Thus, it has good diffusion in corporal tissues, interstitial and intracellular fluids, coming through the blood brain barrier into the amniotic fluid, aqueous humor, lymph tissue, purulent bronchial secretions and fluids (Gobernado, 2003). In pharmacokinetics studies, due to the almost undetectable protein binding, the obtained plasma values represent almost all FOS available at a given moment (Zozaya *et al.*, 2008).

FOS has been shown to exert a time dependant microbial growth inhibition (Sauermann *et al.*, 2005). Thus, it has been speculated that its optimal bactericidal effect can be obtained at three to four times the concentration that inhibits 90% (MIC_90_) of bacterial isolates (Pfausler, 2004) and not necessarily linked to high maximum plasma concentration (C_max_) values (McKellar *et al.*, 2004).

Mazzei *et al*. (2006) have also described a postantibiotic effect (PAE) of 3.4-4.7 h. It is important to note that drugs acting by concentration peak (C_max_/MIC) and antimicrobial dependent AUC/MIC concentration have higher PAE, such as aminoglycosides and ciprofloxacin which have PAE of 2 to 6 h in Gram negatives. The b-lactams do not have PAE in Gram negatives and it is only 2 h in Gram positives. Then, considering the PAE of other drugs, FOS can be considered to have a significant PAE (Labarca, 2002).

### Chemical structure

FOS is a propionic acid derivative which corresponds to the formula of an epoxide. The simple water-soluble molecule is similar to phosphoenolpyruvate (PEP). It has only three carbon atoms and no nitrogen. The antibacterial activity is due to the epoxy bond (Gobernado, 2003).

**Fig. 1:**
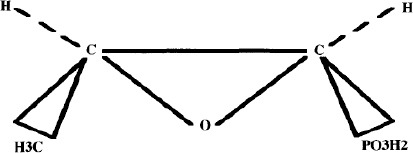
Fosfomycin chemical structure.

The molecule has a number of chemical characteristics which are not typically observed in organo phosphorous compounds. On one hand, it is formed by an epoxy group to which the negatively charged phosphoric group binds and which is decisive for its antibacterial action. On the other hand, it presents a direct bond between the carbon and phosphorus without an oxygen intermediate bridge, as is usual in organo phosphorus compounds (Baron and Drugeon, 1985) (Fig. 1). Its molecular weight is almost the lowest of all the antimicrobials (138 ’1) (Moritz, 1986; Neuman, l990; Gutiérrez *et al.*, 2008), which added to its low protein binding, favors the spread of the drug to tissues and fluids.

FOS tends to form salts easily due to its acidic nature. Its chemical structure is presented in different salts: disodium salt is used for IV and SC administration, while trometamol salt (tromethamine [trihydroxymethyl aminomethane]) and the calcium salt are used for oral administration (Escolar Jurado *et al.*, 1998). Disodium and calcium salts, which are parenterally and orally used, respectively, are obtained by substituting the two hydrogen atoms of the phosphoric radical by two atoms of sodium and one of calcium.

**Fig. 2:**
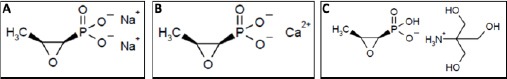
Fosfomycin different salts. (A): Disodium FOS, (B): Calcium FOS and (C): Trometamine FOS. Chemicals properties make FOS a peculiar antibiotic and substitutions of its H atoms by other radicals (Na^+1^ or Ca^+2^) gives rise to the different salts.

Trometamol salt, available since 1990 (Gudiol, 2007) and commercially available for oral use, is obtained by adding a molecule of tromethamine to the phosphoric radical. Tromethamine (tris-hydroxymethyl-aminomethane) is a synthetic buffer for short term use (Gomis *et al.*, 1992), which leads to a molecular weight of FOS that is nearly the double of the original drug, without contributing or interfering with its antibacterial action. Figure 2 shows FOS different salts.

### Spectrum of action

FOS has bactericidal activity against Gram positive and Gram negative bacteria (Mata *et al.*, 1977; Gobernado, 2003) and when compared to penicillins and semi-synthetic cephalosporins, it has a broader spectrum of action (Mata *et al.*, 1977).

FOS bactericidal effect is fast which has been demonstrated by laboratory assays, such as turbidity reduction in liquid culture media and colony reduction on solid media passes (Rodicio *et al.*, 1978; Schmid, 1979; Schmid, 1980; Carlone *et al.*, 1982; Schmid, 1985). Minimum inhibitory concentration (MIC) and Minimum bactericidal concentration (MBC) values are similar to the majority of gram-positive and gram-negative bacteria, being lower when incubated under anaerobic conditions, probably reflecting a lower FOS transport through the cell membrane under these conditions (Inouye *et al.*, 1989; Hamilton-Miller, 1992).

In intensive productions (poultry and swine production), FOS is used for the treatment of infections caused by sensitive Gram positives and Gram negatives germs, such as *Salmonella sp*., *Escherichia coli*, *Pasteurella sp*., *Staphylococcus sp*., *Streptococcus sp*., *Haemophilus sp*., *Klebsiella sp*. (good activity) and *Pseudomona sp*. (moderate activity). Its activity against *Listeria*, *Leptospira*, *Clostridium spp. and Vibrio spp*. is moderate. It is not active against bacteroids (García-Rodríguez, 1984), *Mycobacterium spp., Leggionella spp., Borrelia spp*., and, naturally, against bacteria without cell wall such as *Coxiella burnetii, Rickettsia, Chlamydia, Mycoplasma* and *Ureaplasma*, which are insensible to FOS.

FOS spectrum of action is shown in Table 1. MICs for microorganisms most commonly found in animals, for which FOS was used in their treatment are in the range of 0.25-0.5 g/mL (Fernández *et al.*, 1995) (*Streptococcus spp*., *S. aureus, Enterococcus sp., E. coli*, among others).

**Table 1:** Fosfomycin spectrum of action.

	Good Activity MIC < 16 mL/L	Moderate Activity MIC < 16-64 mL/L	Without Activity MIC < 64 mL/L
Aerobic Gram-positive bacteria
	*Staphylococcus aureus*
	*Staphylococcus epidermidis*
	*Streptococcus pyogenes*	*Staphylococcus haemolyticus*	Other *Staphilococcus spp.*
	*Streptococcus viridans*	*Staphylococcus agalactiae*	*Mycobacterium spp.*
	*Streptococcus pneumoniae*	*Listeria monocytogenes*	*Nocardia sp.*
	*Streptococcus (groups C-F-G)*
	*Enterococcus faecalis*
	*Enterococcus faecium*
Aerobic Gram negative bacteria	-	-
			*Moraxella spp.*
			*Bordetella spp.*
			*Legionella spp.*
Facultative aerobic - anaerobic Gram-negative bacteria
	*Histophilus somni*
	*Escherichia coli*
	*Klebsiella pneumoniae*
	*Serratia spp.*
	*Citrobacter spp.*		*Corynebacterium spp.*
	*Proteus mirabilis*		*Brucella spp.*
	*Proteus vulgaris*
	*Salmonella spp.*
	*Shigella spp.*
	*Aeromonas spp.*
	*Yersinia enterocolitica*
Microaerophilic bacteria	*Campylobacter jejuni*
Anaerobic Gram-negative bacteria
	*Peptococcus spp.*		*Mycobacterium spp.*
	*Fusobacterium spp.*		*Bacteroides*
Gram-negative, without cell wall
			*Coxiella burnetti (Ae)*
			*Rickettsia spp. (Ae)*
			*Chlamydia spp. (Ae)*
			*Mycoplasma spp. (FAA)*
			*Ureaplasma spp. (FAA)*

A= Aerobic

FAA = Facultative aerobic anaerobic

Note that these microorganisms are in the first column of Table 1 that represents species for which FOS has a good *in vitro* activity. FOS has a fast bactericidal effect against a broad spectrum of animal and human pathogens.

### Mechanism of action

FOS penetrates bacteria by two systems of permeases, one that transports L α glycerol phosphate, and other, which is inducible and takes D-glucose-6-phosphate inside the bacterial cytoplasm (Popovic *et al.*, 2010). FOS acts in proliferating bacteria by inhibition of cell wall and early murein/peptidoglycan synthesis (Kahan *et al.*, 1974).

It inhibits an initial step in peptidoglycan synthesis (Mensa *et al.*, 1994), which is triggered by an analog of FOS (Kahan et al., 1974; Popovic *et al.*, 2010), uridine diphosphate N-acetyl-glucosamine-enol-pyruvyl-transferase and its co-enzyme, phosphoenol-pyruvate (PEP).

FOS acts on bacteria in the growth phase without interfering with the reactions requiring PEP in animal cells. This is because, in animals, enzymatic attack occurs at a different place from PEP and the enzyme does not recognize FOS as a substrate. FOS inhibits the binding of PEP to N-acetylglucosamine. For wall synthesis, the group-O-PO3H2 of PEP is separated, binding the pyruvate C2 to the oxygen of an N-acetylglucosamine.

**Fig. 3:**
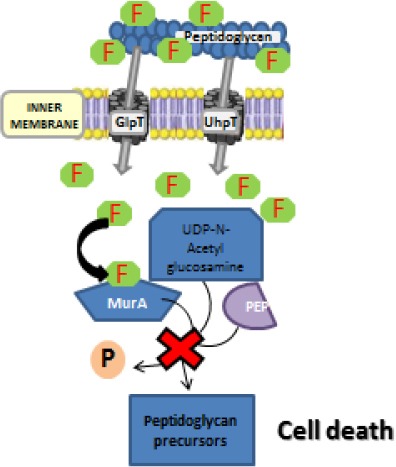
FOS (F) is transported inside the cell by glycerol-3-phosphate transporter (GlpT) and glucose-6-phosphate transporter (UhpT) blocking the UDP-GlcNac-3-O-enolpyruvate synthesis by mimicking the original substrate of UDP-GlcNAc enolpyruvyl transferase (MurA), phosphoenolpyruvate (PEP), and in the process avoiding cell wall synthesis and leading to cell death.

However, in eukaryotic cells, the oxygen remains attached to C2, separating only the phosphate PO3H2. FOS has in its molecule the -OCP-sequence, which is different from the -COP sequence of PEP. This fact explains the high selectivity of FOS, which inhibits the use of PEP in the cell wall synthesis (where the enzyme cleaves OL binding) and not in the metabolism of eukaryotic cells (where enzymes break the union OP). Figure 3 shows FOS mechanism of action.

FOS inhibits cell wall synthesis due to its analogy with uridine diphosphate N-acetyl-glucosamine-enol-pyruvyl-transferase. PEP is the coenzime of the reaction. However, FOS does not interfere with the reactions requiring PEP in animal cells. In animals, enzymatic attack occurs at a different place from PEP and the enzyme does not recognize FOS as a substrate.

### EFFECT OF THE ASSOCIATION WITH OTHER ANTIBIOTICS

Due to its mechanism of action in the first steps of cell wall production, FOS can act synergistically with other antibiotics, especially those which inhibit the late stages in the cell wall synthesis (Gudiol, 2007). It shows a synergistic partnership with other antimicrobials, mainly, with beta-lactams, aminoglycosides, chloramphenicol, tetracycline, erythromycin, cotrimoxazole and quinolones (Salhi *et al.*, 1986; Damaso *et al.*, 1990; Ilender, 1998).

In association with penicillin it has a synergistic effect on *S. aureus* and *S. pneumoniae*, with ampicillin it is synergic on *S. aureus* and *E. coli* and with cephalosporins, it has synergistic effect on *S. aureus* and *P. aeruginosa*. Furthermore, synergism with vancomicin has been demonstrated on *S. aureus* and *S. epidermidis*, with imipenem on *S. epidermidis and K. pneumoniae*, with rifampicin on *S. epidermidis* and *E. faecalis*, with ciprofloxacin, on *S. aureus*, *S. epidermidis* and *E. faecalis* with streptomycin it is synergic on *E. coli* and has a synergistic-additive effect on *S. aureus*, and *P. aeruginosa* (Gobernado, 2003). Table 2 shows FOS synergistic partnership with other antimicrobials.

**Table 2:** Effect of Fosfomycin in association with other antibiotics.

FOS associated with	Microorganism	Effect
Penicillin
	*Staphylococcus aureus*
	*Staphylococcus pneumoniae*	Synergistic
Ampicillin		
	*Staphylococcus aureus*
	*Escherichia coli*	Synergistic
Cephalosporins		
	*Staphylococcus aureus*
	*Pseudomonas aeruginosa*	Synergistic
Vancomicin		
	*Staphylococcus aureus*
	*Staphylococcus epidermidis*	Synergistic
Imipenem		
	*Staphylococcus epidermidis*
	*Klebsiella pneumoniae*	Synergistic
Rifampicin		
	*Staphylococcus epidermidis*
	*Enterococcus faecalis*	Synergistic
Ciprofloxacin		
	*Staphylococcus aureus*
	*Staphylococcus epidermidis*
	*Enterococcus faecalis*	Synergistic
Streptomycin		
	*Escherichia coli*	Synergistic
Streptomycin
	*Staphylococcus aureus*
	*Pseudomonas aeruginosa*	Synergistic-Additive

FOS acts synergistically with antibiotics which inhibit the late stages of cell wall synthesis. The pathogens mainly affected by this synergistic effect are *S. aureus*, *S. epidermidis*, *K. pneumoniae*, *P. aeruginosa* and *E. coli*.

### RESISTANCE

Bacterial resistance to FOS has been related to transport alteration through cell wall, target alteration and, rarely, to enzymatic breakage (Gobernado, 2003). Besides this natural resistance, acquired resistance associated with transport or chromosomic alterations has also been reported (Damaso *et al.*, 1990). Extrachromosomal resistance, governed by plasmids, has also been described (Obaseiki-Ebor, 1986; Villar *et al.*, 1986). Castañeda-García *et al*. (2013) considers three different possible mechanisms leading to FOS resistance: a) reduced permeability to FOS, b) modification of the antibiotic target MurA (UDP-GlcNAc enolpyruvyl transferase), c) antibiotic modification.

Chromosomal resistance is manifested by the production of the enzyme FOS glutathione S-transferase, which inactivates the antibiotic by producing a bond between glutathione and FOS (Arca *et al.*, 1990). The enzyme is located in the periplasmic space. This kind of resistance has been described in both gram-positive and gram-negative bacteria (Venkateswaran and Wu, 1972; Kurashige *et al.*, 1975; Cordaro *et al.*, 1976; Gershanovich *et al.*, 1980; Hardisson *et al.*, 1984; Mlynarczyk *et al.*, 1985; Ravdonikas *et al.*, 1988; Corso *et al.*, 1998).

Transferable plasmid resistance is conditioned by permeability of the cell membrane alteration and enzymatic modification of the antibiotic (Llaneza *et al.*, 1985). Another described mechanism of resistance is FOS inactivation by opening of the bond between carbon and phosphorus by the C-P-lyase enzyme (Quinn, 1989).

In almost all susceptible bacterial populations, FOS single step resistant spontaneous mutants exist with a high frequency (1/104 to 1/106). This resistance is due to the inability of FOS to penetrate the bacterial cell by a deficiency of transport systems, such as L-alpha-glycerol phosphate and D-glucose-6-phosphate (Baron and Drugeon, 1985; Damaso *et al.*, 1990). There is no evidence of cross-resistance to any other antibiotic or chemotherapeutic (Baron and Drugeon, 1985; Damaso *et al.*, 1990; Patel *et al.*, 1997; Gobernado, 2003; Gudiol, 2007; Gutiérrez *et al.*, 2008).

Similar to other antibiotics, shortly after the beginning of FOS commercialization, the concern for the evolution of resistance started. However, after several studies conducted *in vitro* from the ’70s to the present using human isolated bacteria demonstrated that the activity against common pathogens causing infections in which this antibiotic is indicated has not significantly changed (Gobernado, 2003).

FOS natural bacteria resistance may be due to transport alteration through cell wall, target alteration and enzymatic breakage. Acquired resistance is also associated with transport chromosomic alteration. Extrachromosomal resistance, governed by plasmids, also has been described. Three other different possible mechanisms leading to FOS resistance are the reduction of permeability, modification of the antibiotic target MurA and antibiotic modification.

### OTHER EFFECTS

In addition to the antibacterial activity, FOS has other properties, such as inhibition of the adhesion of bacteria to epithelial cells, exopolysaccharide biofilm penetration, immunomodulation, phagocytosis promotion and protection against the nephrotoxicity caused by other drugs (Gobernado, 2003).

### Bacterial adhesion

While some antibiotics at concentration under the MIC induce the formation of filamentous bacteria, favoring adherence to the urothelial cells, FOS reduces this phenomenon. In addition to its anti-adhesive effect, at concentrations under the MIC, FOS also decreases hemolysin production and the hydrophobicity of *E. coli*, which is important in the prophylaxis and treatment of repeated urinary tract infections (Gismondo *et al.*, 1994).

### Biofilms

For most antibiotics it is very difficult to penetrate the infected exopolysaccharide biofilms that are formed on catheters, prosthetics, kidneys and other organ sites. FOS, macrolides and fluoroquinolones penetrate acceptably into biofilms, and the association of FOS with macrolides or quinolones improve the penetration. Furthermore, it has been shown that FOS produces significant alterations in cell morphology and in the outer membrane of *P. aeruginosa* incorporated into biofilms (Kumon *et al.*, 1995; Moden *et al.*, 2002).

### Phagocytosis

It has been shown that FOS, at concentrations equal to or above the MIC, kill microorganisms located within phagocytes (Traub and Spohr, 1983). An increased neutrophil bactericidal activity has been described in the presence of FOS (Krause *et al.*, 2001). Studies in rabbits have shown that somatic antibody titers in flagellar bacteria exposed to FOS- immunized animals were higher than those observed in animals immunized with bacteria not exposed to the drug (Viano *et al.*, 1979). *In vitro*, it has been shown that FOS promotes migration and chemotaxis of polymorphonuclear phagocytes, probably by inhibiting respiratory enzymes, the presence of inactive metabolites of drugs and Adenine monophosphate-Guanosine monophosphate (APM-GMP) cycle alteration (De Simone *et al.*, 1980).

### Immunomodulation

Numerous immunomodulatory effects of FOS have been reported. It has been shown to inhibit human lymphocyte proliferation and to decrease the release of IL-2, probably by blocking cell division T (Morikawa *et al.*, 1993). It has been demonstrated the inhibition of the B cell proliferative response stimulated by *S. aureus* and the production of immunoglobulins without altering the expression or activation of antigens, such as CD25 and CD71 (Morikawa *et al.*, 1996). Some authors consider that FOS modifies the acute inflammatory response due to decreased synthesis of TNF-α, IL-1 α, IL-1β, the receptor antagonist of IL-1 and granulocyte colony stimulating factor (Morikawa *et al.*, 1996; Matsumoto *et al.*, 1999). It has also been shown that the sensitivity of cells to TNF-β increases in the presence of FOS (Ishizaka *et al.*, 1998). FOS has been shown to suppress LB4 production in neutrophils and to decrease the expression of IL-8 (Honda *et al.*, 1998). The antiallergic property was based on its ability to suppress, *in vitro*, histamine release (Ida *et al.*, 1987). Studies on a murine experimental model have confirmed the overall favorable immunomodulatory effect of FOS (Matsumoto *et al.*, 1997).

### Protection against nephrotoxicity and ototoxicity

Studies in animals and humans have shown that the concomitant use of FOS with drugs that cause nephrotoxicity and ototoxicity, such as cisplatin, cyclosporine (antitumor) (Sack *et al.*, 1987; Suzuki *et al.*, 1991; Nakamura *et al.*, 1998), aminoglycosides, vancomycin, amphotericin B and polymyxin (antibiotics) (Inouye *et al.*, 1982; Morin *et al*.; 1984) protects against the undesirable effects of the other drugs.

The great variety of effects, in addition to its antibacterial capacity, makes FOS a multifaceted drug.

### PHARMACODINAMICS

In all species, important differences in the bioavailability (F) have been found after oral administration in relation to the various derivatives of FOS salts, such as disodium FOS (41-85%), calcium FOS (20%) and trometamol FOS (34-41%) (Segre *et al.*, 1987; Patel *et al.*, 1997). Furthermore, the IM administration of disodium FOS offers a more predictive route of dose absorption than PO administration. This difference may be associated with two facts: a) absorption from the gastrointestinal tract is a saturable process associated with the phosphate system and b) there is degradation of disodium FOS in acid gastric pH (Gutiérrez *et al.*, 2008). The IM route is more predictive for dose absorption. Nevertheless, PO administration is useful for the treatment of intestinal infections, especially when the drug has poor bioavailability.

There are differences in the bioavailability of FOS after IM and PO administrations, which are related with the type of salts used.

## PHARMACOKINETICS

### Routes of administration

For PO administration, FOS is used as a calcium salt, whereas IV, IM and SC routes require the more water-soluble disodium salt. FOS-tromethamine salt is highly hydro-soluble and has good bioavailability after oral administration (Patel *et al.*, 1997; Popovic *et al.*, 2010).

### Absorption

After PO administration, absorption of FOS occurs throughout the digestive tract. However, it is higher in the duodenum.

IM administration of disodium FOS shows fast and complete absorption. Absorption after PO administration has demonstrated to be variable and to differ between species. In mice, rats and dogs the range of absorption of the administered dose is of 50-80%, whereas in humans, its absolute bioavailability is 37-40%.

Furthermore, differences are also observed, depending on whether the calcium salt or Trometamol is administered. Calcium salt absorption is not affected by the presence of food, although its bioavailability (F%) is lower (20-30). Tromethamine salt should be administered on empty stomach since the presence of food reduces the rate of absorption and, therefore, its F. However, F (40) is higher than that found with the calcium salt. FOS absorption occurs through a saturable carrier-mediated mechanism and by nonsaturable passive diffusion, as determined by *in situ* and *in vivo* experiments in rats (Ishizawa *et al.*, 1991).

It is suggested that the carrier-mediated transport is more important for absorption, especially at concentrations of less than 1 mM FOS. Studies carried out in rats, rabbits and humans show that the phosphate transport might be important for the intestinal absorption of this antibiotic.

Relatively small molecules which include phosphate within their structure may be the substrates for the sodium-ion-dependent transporter, enhancing the intestinal absorption (Tamai and Tsuji, 1996).

### Distribution

As previously described, low protein binding (<0.5%) along with its low molecular weight and water solubility, allows good diffusion of FOS in interstitial fluid and tissues.

### Cerebrospinal fluid (CSF)

No animal studies have been conducted regarding the concentration of FOS in CSF. In humans, it has been found that it readily crosses the blood brain barrier, diffusing to CSF (Gallego *et al.*, 1971). Several studies have shown that FOS is useful in the treatment of meningitis caused by *S. pneumoniae*, *Staphylococcus*, *E. coli* and other Gram negative sensitive bacilli (Drobnic *et al.*, 1997; Falagas *et al.*, 2008) when it is IV administered (1-12 g/day). FOS concentrations in CSF were determined to be 27.7% of that obtained in blood, lower than the concentrations found for chloramphenicol (32%), but higher than the values found for penicillin (7.9%) and ampicillin (15.9 %) (Sicilia *et al.*, 1981). Furthermore, several authors have found that the penetration of FOS in CSF is higher (300%) in inflamed meninges compared to non-inflamed (Boulard *et al.*, 1983; Pfeifer *et al.*, 1985; Kuhnen *et al.*, 1987).

### Interstitial fluid

In humans, it has been demonstrated that FOS reaches values between 34-43% of plasma concentrations in interstitial fluid and cellular subcutaneous tissue in patients with cellulite and diabetic foot syndrome after an IV infusion (Legat *et al.*, 2003). In addition, when IV administered, it has been demonstrated to penetrate the interstitial fluid of patients with burns (Koh *et al.*, 1986) and to reach the muscular interstitial fluid (Joukhadar *et al*. 2003). In animals, the only known studies were performed by Fernández Lastra *et al*. (1987) in interstitial fluid of rabbits and by Soraci *et al*. (2011c) in the fluid lining the bronchial epithelial of pigs. Fernandez Lastra *et al*. (1987) observed that after IV administration the half-life of FOS in interstitial fluid is 1.9 h, either with single or multiple dosages. After IM administration (15/mg/kg b.w.) of disodium FOS, Soraci *et al*. (2011a) showed that the drug reaches concentrations above the MIC90 of pathogens such as *Streptococcus*, for more than 8 h in bronchial epithelial lining fluid. These results demonstrate that FOS is useful for treating diseases caused by extracellular microorganisms that are involved in swine respiratory disease.

### Placenta

No animal studies regarding FOS passage through placenta have been conducted. It has been demonstrated in humans that after IM administration at a dose of 1 g, FOS crosses the placental tissue, and reaches fetal maternal blood at ratios of 0.9; 0.27 and 0.68 at 30, 90 and 120 minutes (Ferreres *et al.*, 1977). Although it is apparent that the drug is safe to be administered during pregnancy, trometamol FOS has not been approved in all European countries for it use in pregnant women (Raz, 2012). Studies in animals have not shown trometamol FOS teratogenicity (Ferreres *et al.*, 1977). In contrast to prolonged therapy the administration as a single dose in pregnancy reduces the risk to the fetus. However, it is recommended to be used in pregnancy only in cases where favorable risk/benefit is deemed.

### Aqueous and vitreous humor

Most studies were performed in humans (Radda *et al.*, 1985; Adenis *et al.*, 1987; Robert and Tassy, 2000). Only a pilot study conducted in rabbits (Adenis *et al.*, 1987) is available. In all cases, it was found that FOS reaches concentrations which are enough to inhibit most pathogens that cause endophthalmitis after IV infusion. Its use in patients with cataracts (Forestier *et al.*, 1996) has also been shown.

### Bone

In humans it has been demonstrated that FOS penetrates into the cortical and cancellous bone area after IV administration. High concentrations have been found in both zones (15% of plasma concentration) (Sirot *et al.*, 1983; Meissner *et al.*, 1989). An experimental study in rats has been conducted using 200 mg of FOS, SC administered, in patients with osteomyelitis caused by *Pseudomonas aeruginosa*. This study concludes that FOS reaches good concentrations in bone and that concentrations are higher in infected bones of rats with chronic osteomyelitis (Fe Marques, 1994).

### Colostrum and milk

A small proportion of FOS is eliminated by milk and colostrum. Fernandez Paggi *et al*. (2010) studied the distribution of disodium FOS in sow colostrum after the IM administration of 15 mg/kg b.w. in pigs during the peri-partum. FOS distribution in breast fluid is low and of short-term (8 h). Therefore, it could be administered to the sow during lactation without side effects in the piglets.

### Metabolism and Excretion

FOS has no metabolic transformation (Roussos *et al*, 2009). It is excreted in urine in active form, mainly by glomerular filtration (10% to 60%) without tubular secretion or reabsorption. Thus, its renal clearance is similar to creatinine (Eardley *et al.*, 2006). Although, the excreted amount depends mainly on the administration form, when parenteral administration is employed 85 to 95% of the dose is excreted in urine reaching urinary concentrations in the order of 1000 to 3000 mcg/mL (Roussos *et al.*, 2009). Its high concentration in urine is maintained for at least 36 h. When orally administered, one third of the absorbed amount is excreted in urine and the remaining amount is eliminated in feces.

When trometamol salt is parenterally or orally administered, it shows some biliary unmetabolized elimination (20%) and is actively reabsorbed back to the intestine. This enterohepatic circulation explains the appearance of a secondary serum peak (Segre *et al.*, 1987).

In renal failure, when the glomerular filtration rate is between 20-40 mL/min, it is advisable to administer 75% of the normal dosage, and when it is less than 10 mL/min a dosage reduction to 25 % is recommended.

Bioavailability after parenteral administration corresponds to a two compartment open model. FOS does not bind to plasma proteins and, therefore, it becomes available as a fully active molecule.

Pharmacokinetic (PK) profiles of the various derivatives of FOS have been described in humans (Kirby, 1977; Segre *et al.*, 1987; Vargas *et al.*, 1987), chickens (Aramayona *et al.*, 1997; Soraci *et al.*, 2011b), rabbits (Fernández Lastra *et al.*, 1987), cows (Sumano *et al.*, 2007), dogs (Gutiérrez *et al.*, 2008), horses (Zozaya *et al.*, 2008) and weaning piglets (Soraci *et al.*, 2011a).

### FOS pharmacokinetics in broiler chickens

Three FOS pharmacokinetic studies have been conducted in broiler chickens (Aramayona *et al.*, 1997, Gutiérrez *et al.*, 2010; Soraci *et al.*, 2011b). Aramayona *et al*. (1997) studied the pharmacokinetics of FOS in chickens after a single IV dose (10 mg/kg b.w.). Gutiérrez *et al*. (2010) studied the kinetics of FOS after IV administration (10, 20, 40 and 80 mg/kg b.w.) and PO administration (10, 20, 40 and 80 mg/kg b.w.). Soraci *et al*. (2011b) studied the kinetics of disodium FOS after IV (40 mg/kg b.w.) and IM (10 mg/kg b.w.) administration and calcium FOS after PO administration (40 mg/kg b.w.). The authors found an increased bioavailability of FOS when administered IM (82%) compared to PO administration (39.3%). The volume of distribution determined by Soraci *et al*. (2011b) for FOS IV administration (231 mL / kg), is comparable to that found by Aramayona *et al*. (1997) (575 mL / kg) and by Gutiérrez et al. (2010) (250-220 mL/kg). The elimination half-life of FOS after IV bolus administration (1.4 h; Soraci *et al.*, 2011b, 1.8 h, Aramayona *et al.*, 1997) is similar to that observed after PO (1.3 h) and IM (1.1 h) administration. The clearance is comparable to the percentage of glomerular filtration rate (2.1 ml. min-1.kg^-1^) (Soraci *et al.*, 2011b) and similar to that reported by Aramayona *et al*. (1997) (2.65 to 3.69 ml. min-1.kg^-1^).

### FOS pharmacokinetics in rabbits

There are few pharmacokinetic studies conducted in rabbits. In 1978, Yaginuma *et al.*, studied the pharmacokinetics of IV sodium salt preparation of FOS in this species. In 1986, Fernandez Lastra *et al*. studied the linearity of the pharmacokinetics of FOS in serum and interstitial tissue fluid in rabbits, after administration of doses of 20, 30 and 60 mg/kg b.w. of the antibiotic by SC implantation of spiral steel cages. The elimination half-lives of FOS ranged between 1.16 and 1.57 h. In 1987, Fernández Lastra *et al.*, studied FOS levels in serum and tissue interstitial fluid in a multiple dosage regimen in rabbits, after the administration of a single dose of 60 mg/kg b.w. and during a multiple dosage regimen of 60 mg/kg/6h over three days. The elimination half-life of the drug from the systemic circulation after a single dose had a value of 1.6 h, and was not significantly different from the value found for the same parameter in the multiple dosage regimens.

### FOS pharmacokinetics in cattle

There is only one pharmacokinetic study of FOS in cattle. It was performed in 2007 by Sumano *et al*. They have studied the IV and IM pharmacokinetics of a single-daily dose of disodium FOS (20 mg/kg/day), administered for 3 days. The calculated concentrations at time zero and maximum serum concentrations were 34.42 and 10.18 µg/mL (T_max_: 2.98 h), respectively. The elimination half-life of the drug remained unchanged during the 3 days (= 1.33 +/- 0.3 h for the IV route and = 2.17 +/- 0.4 h for the IM route). Apparent volumes of distribution suggest moderated distribution out of the central compartment (V (d_area_) = 673 mL +/- 27 mL / kg and V (dss) = 483 +/- 11 mL/kg). Bioavailability after IM administration was 74.52%.

### FOS pharmacokinetics in dogs

In 1978, Yaginuma *et al.*, studied the pharmacokinetics of an IV preparation of disodium FOS salt in dogs. Gutiérrez *et al*. (2008) also studied FOS pharmacokinetics in mongrel dogs. Nevertheless, they studied the variables after the administration of buffered disodium FOS by IV, IM, SC and PO routes at 40 and 80 mg/kg/day for three days. A non-accumulative kinetic behavior was observed after three days with both doses and most pharmacokinetic variables remaining unaltered. The authors concluded that useful plasma concentrations can only be achieved after the SC injection of 80 mg/kg b.w. every 12h, having a C_max_=18.96+/-0.3 µg/mL; a T_1/2β_=2.09+/-0.06 µg/mL and a bioavailability of 84-85%.

### FOS pharmacokinetics in horses

In 2008, Zozaya *et al*. studied FOS pharmacokinetic parameters in horses after the administration of disodium FOS at 10 mg/kg b.w. and 20 mg/kg b.w. by IV, IM and SC routes. Bioavailability after the SC administration was 84 and 86% for the 10 mg/kg b.w. and the 20 mg/kg b.w. dose, respectively. It was concluded that clinically effective plasma concentrations might be obtained for up to 10 h administering 20 mg/kg b.w. SC.

### FOS pharmacokinetics in pigs

At present the only documented clinical experience of the use of FOS in pigs are the studies of Soraci *et al*. (2011a) and Pérez *et al*. (2012b). Soraci *et al*. (2011a) studied the pharmacokinetics and the bioavailability of disodium FOS in post-weaning piglets after IV and IM administration of 15 mg/kg b.w. After IV administration, the area under the FOS concentration:time curve in plasma was AUC_(0-12)_ of 120.00 ± 23.12 μg h/mL and the volume of distribution (Vd) of 273.00 ± 40.70 ml/kg.

Plasma clearance was of 131.50 ± 30.07 ml/kg/h and a T_1/2_ of 1.54 ± 0.40 h. Peak serum concentration (C_max_), T_max_, AUC_(0-12)_ and bioavailability for the IM administration were 43.00 ± 4.10 μg/ml, 0.75 ± 0.00 h, 99.00 ± 0.70 μg h/ml and 85.5 ± 9.90%, respectively. Pérez *et al*. (2012b) studied the pharmacokinetics and the bioavailability of calcium FOS in post-weaning piglets after PO administration of 30 mg/kg b.w. The T1/2 was of 1.80 ± 0.89 h. C_max_, T_max_ and bioavailability were 3.60 ± 0.96 µg/mL, 3.00 ± 0.00 h and 20.0 ± 1.85 %, respectively. The area under the FOS concentration:time curve in plasma AUC(0-∞) was 45.48 ± 9.20 µg h/mL. Table 3 shows a summary of the pharmacokinetics parameters of FOS in animal species. For PO administration, FOS is used as calcium and tromethamine salts, whereas for IV, IM and SC administrations FOS is used as the more water-soluble disodium salt. After PO administration, absorption occurs throughout the digestive tract. Disodium salt presents a fast and complete absorption (IM), which occurs through both a saturable carrier-mediated mechanism and a nonsaturable passive diffusion process.

**Table 3:** Fosfomycin pharmacokinetics parameters in animals

SPECIES	BROILER CHICKENS				PIGS			CATTLE			DOGS			HORSES		
AUTHOR	Aramayona *et al.* (1997)	Soraci *et al.* (2011b)			Soraci *et al.* (2011a)		Pérez *et al.* (2012b)	Sumano *et al.* (2007)		Gutiérrez *et al*. (2008)								Zozaya *et al*. (2008)					
DETERMINATION METHOD	Microbiologic DL: 0.5 ppm	HPLC MS/MS DL: 0.1 ppm			HPLC MS/MS DL: 0.1 ppm		HPLC MS/MS DL: 0.1 ppm	Microbiologic DL: 0.4 ppm		Microbiologic DL: 0.4 ppm								Microbiologic DL: 1.05 ppm					
FOS FORMULATION	Disodium	Disodium (IV, IM) and Calcium (PO)			Disodium		Calcium	Disodium		Disodium								Disodium					
ADMINISTRATION ROUTE	IV	IV	IM	PO	IV	IM	PO	IV	IM	IV		PO		IM		SC		IV		IM		SC	
DOSE (mg/kg)	-	40	40	10	15	15	30	20	20	40	80	40	80	40	80	40	80	10	20	10	20	10	20
F (%)	-	-	39.3	81.75	-	85.50	20.00	-	74.52	-	-	30	29	41	43	84	85	-	-	38.00	58.00	84.00	86.00
AUC (µg.h/mL)	-	318	125.00	65.10	120	99.00	45.48	78.35	56.49	92.54	176.26	22.50	48.72	36.41	82.12	78.25	143.14	307	410	115.00	224.00	249.00	315.00
C_max_ (µg/mL)	-	-	29.79	20.70	-	43.00	3.60	-	10.18	-	-	5.20	10.84	9.61	21.71	9.46	13.96	-	-	24.00	46.00	55.00	72.00
T_max_ (h)	-	-	2.00	0.80	-	0.75	3.00	-	2.98	-	-	2.04	1.75	1.08	1.19	2.63	2.51	-	-	2.37	2.46	3.32	3.24
T_1/2_	1.86	1.39	-	-	1.54	-	1.80	2.50	2.17	1.28	1.30	2.18	2.18	1.54	1.55	2.06	2.09	1.33	1.34	1.54	1.57	3.43	3.46
Vd (mL/kg)	575	231	-	-	273	-	-	483	-	690	700	-	-	-	-	-	-	215	220	-	-	-	-
Cl (mL/kg/h)	3.12	115	-	-	131.5	-	-	11.20	-	14.20	14.90	-	-	-	-	-	-	16.00	24.00	-	-	-	-

Low protein binding, along with its low molecular weight and water solubility, allow good diffusion into interstitial fluid and tissues. It has no metabolic transformation. Therefore, it is excreted in urine in active form by glomerular filtration. PK profiles of the various derivates of FOS have been described in humans, chickens, rabbits, cows, dogs, horses and weaning piglets with the differences and similarities mentioned above.

### Treatment protocols for different species

It is important to note that FOS can be used both therapeutically and prophylactically and different protocols of use have been suggested in several species.

### Broiler chickens

Aramayona *et al*. (1997) suggest that PO administration of FOS in drinking water at a dose of 150 pg/mL for 5 consecutive days provides potentially therapeutic concentrations of the drug in chickens. Gutiérrez *et al*. (2010) suggest that useful serum concentrations of disodium FOS to treat outbreaks of susceptible *E. coli* require an initial loading dose of 40 mg/kg b.w., followed by an ad libitum medication of 40 mg/kg b.w. 8 h later (80 mg/kg per d). Soraci *et al*. (2011b) concluded that effective plasma concentrations of FOS for sensitive bacteria can be obtained following PO and IM administration. They suggest a useful dose of 10 mg/kg b.w. of disodium FOS by IM administration. After PO administration of calcium FOS at a dose of 40 mg/kg b.w. and an IM dose of disodium FOS at 10 mg/kg b.w., authors consider that there is an insufficient therapeutic efficacy *in vivo* in a single dose at an interval of 24 hrs.

### Rabbits

Fernandez Lastra (1986, 1987) has found good results using doses between 20-60 mg/kg b.w, after SC administration (single and multiple dose dosage).

### Cattle

Sumano *et al*. (2007) suggest that clinically effective plasma concentrations of disodium FOS could be obtained for up to 8 h following IV administration and for approximately 10 h after IM injection of 20 mg/kg b.w., for susceptible bacteria. In addition to residue studies in milk and edible tissues, a series of clinical assessments, using FOS at 20 mg/kg b.w., are warranted before this antibacterial drug can be considered for use in cattle.

### Dogs

Gutiérrez *et al*. (2008) concluded that useful plasma concentrations can only be achieved after the SC injection of 80 mg/kg every 12h.

### Horses

Zozaya *et al*. (2008) determined that clinically effective plasma concentrations might be obtained for up to 10 h administering 20 mg/kg b.w. of disodium FOS, SC administered.

### Pigs

Soraci *et al*. (2011a) conclude that effective plasma concentrations of disodium FOS for sensitive bacteria of piglets can be obtained following IV and IM administration of 15 mg/kg b.w. Pérez *et al*. (2012b), determined that effective plasma concentrations of calcium FOS for sensitive bacteria can be obtained following PO administration of 30 mg/kg b.w.

### Chickens

150 pg/mL for 5 consecutive days (drinking water); initial loading dose of 40 mg/kg b.w., followed by an ad libitum medication of 40 mg/kg b.w. 8 h later (80 mg/kg per d); IM (10 mg/kg b.w.), PO (40 mg/kg b.w).

### Rabbits

20-60 mg /kg b.w, SC. Cattle: 20 mg/kg b.w., IM. Dogs: 80 mg/kg every 12h, SC. Horses: 20 mg/kg b.w., SC. Pigs: 15 mg/kg b.w., IM; 30 mg/kg b.w., PO.

### Pharmacoeconomics

Several studies suggest that a single dose of FOS is cost effective compared to other antibiotics for the treatment of similar infections. However, cost may be increased with repeated dosing (Shrestha and Tomford, 2001; Pullukcu *et al.*, 2007; Popovic *et al.*, 2010).

FOS is cost effective.

### Clinical use

Although the Food and Drug Administration (FDA) has only approved the use of FOS for the treatment of infectious cystitis, it has been used to treat a broad variety of bacterial infections in humans, such as localized peritonitis, brain abscesses caused by *Staphylococcus spp*., *Streptococcus spp*. and *E. coli* (Sauermann *et al.*, 2005), severe soft tissue infections caused by *S. aureus* and *S. epidermidis* and other conditions (Krause *et al.*, 2001; Joukhadar *et al.*, 2003).

In veterinary medicine, FOS is an antibiotic widely used in farms in Argentina, Brazil and Central America, being mainly prescribed in the treatment of infectious diseases of broiler chickens and pigs. Other antibiotics used for this purpose in poultry and pig production are chlortetracycline, oxytetracycline, tiamulin, tylosin, tilmicosin, enrofloxacin, sulfadiazine and penicillin, which are more used than FOS in other countries. In broilers, FOS has been used to treat *E. coli* and *Salmonella spp*. infections (Fernández *et al.*, 1998, 2001, 2002). Particularly in piglets, FOS is indicated to treat a wide variety of bacterial infections (*Haemophilus parasuis*, *Streptococcus suis*, *Pasteurella multocida*, *Bordetella brochiseptica*, *Staphylococcus hyicus*, *Escherichia coli*), associated with stress and/or to different viral diseases (Martineau, 1997).

The use of FOS in dogs has only been suggested based on its low toxicity and potential efficacy (Pickrell *et al.*, 1993; Gutiérrez *et al.*, 2008). Presently, documented clinical experience of the use of FOS in horses (Zozaya *et al.*, 2008) and cattle (Sumano *et al.*, 2007) is not available.

FOS has been used to treat a broad variety of bacterial infections in humans. In veterinary medicine, it is widely used in farms in Argentina, Brazil and Central America, being mainly prescribed in the treatment of infectious diseases of broiler chickens and pigs.

### Toxicity and side effects

The low toxicity and potential efficacy of FOS are the main factors that contribute to its use in humans and animals (Gallego *et al.*, 1974). Side effects are rare and not serious. LD50 in mice (intraperitoneally) is 4 g/kg for the sodium salt and 20 g/kg for calcium FOS (Gallego *et al.*, 1971). In humans, it can occasionally produce loose stools, diarrhea, nausea and vomiting when administered PO. The administration of 2 g per day divided into 4 doses for 28 days in dogs only caused intestinal disbacteriosis, fully recovered within two weeks of completion of treatment (Damaso *et al.*, 1990). It has also been described eosinophilia, thrombocytosis and discrete transaminase elevations. IV infusion may promote the development of hypernatremia or hypokalemia (Baron and Drugeon, 1985). Allergies, anaphylaxis or severe hypersensitivity have not been recorded. A few cases of slight rash or hives which usually did not force discontinuing the treatment have been reported (Damaso *et al.*, 1990). Its lack of teratogenic action for rabbit and mouse, lead to consideration that FOS a safe drug to be administered during infancy and, probably, during pregnancy (Prieto, 1986). Parenteral administration is painful. Thus, the solution is prepared with lidocaine. In humans, induration at the injection site and IV phlebitis have been described.

FOS has low toxicity and side effects are rare and not serious.

### Intracellular penetration

FOS penetration is demonstrated in phagocytic cells, where high concentrations are reached, presenting an intracellular activity close to that of rifampicin (Baron and Drugeon, 1985; Trautmann *et al.*, 1992). Pérez *et al*. (2012a) studied FOS concentrations in respiratory cells (HEP-2). Intracellular concentrations of FOS were analyzed by HPLC MS/MS. Two formulations of FOS were assayed (disodium FOS: 280 and 130 μg/mL; calcium FOS: 130 μg/mL). Concentrations in HEp-2 cells incubated with 280μg/mL of disodium FOS ranged from 0.74 to 2.79μg/mL (T_max_: 12 h). When incubated with the same formulation of FOS at a concentration of 130 μg/mL, intracellular concentrations ranged between 0.31 and 1.60 μg/mL (T_max_: 12 h). Calcium FOS reached intracellular concentrations that varied between 0.46 and 1.11 μg/mL (T_max_: 8 h). FOS concentrations exceeded the MIC_90_ for the most important pathogens in swine respiratory infections (*Streptococcus spp*.; 0.25μg/mL). Therefore, it is apparent that FOS is an alternative drug for the treatment of intracellular respiratory infections in pigs.

Martínez *et al*. (2011) have studied FOS penetration in cell culture lines and evaluated the interactive effect of deoxinivalenol (DON) on the penetration of the antibiotic. The results showed that intracellular antibiotic concentrations in HEp-2 cells incubated with 130 ppm of calcium FOS oscillated between 0.4 and 1.12 mg/ml with a T_max_ of 8 h. When HEp-2 cells were incubated with FOS and DON, a significant variation was not observed in the cellular penetration of the antibiotic, according to the C_max_ (1.10 ppm) and T_max_ (12 h). Authors concluded that the presence of the mycotoxin would not alter the cellular distribution of FOS in pigs.

Pérez *et al*. (2013a) studied the penetration of FOS in an *in vitro* model of intestinal cells (IPEC-J2 cells). Cells cultures were subjected to 580 µg/mL of calcium FOS. Intracellular concentrations of the antibiotic were analyzed by HPLC MS/MS and they ranged from 23.48 to 45.81 µg/mL (T_max_: 4 h). FOS concentrations exceeded the MIC_90_ for the most important pathogens in swine intestinal infections (*Escherichia coli*: 0.50 µg/mL, *Salmonella enterica subsp. enterica*: 4 µg/mL). Therefore, it is apparent that FOS is an alternative choice for the treatment of intestinal infections in pigs.

Martínez *et al*. (2012) cultured intestinal explants from the jejunum of pigs and applied the model to study the intracellular penetration of FOS in the presence or absence of DON. The results suggest that there was no statistically significant difference in the intracellular concentration of FOS between explants incubated with 580 ppm FOS and explants incubated with 580 ppm FOS and 1ppm DON. The C_max_ was 12 ppm and the T_max_ was 2 h. Only 2 % of the antibiotic is intracellularly accumulated and the intracellular concentration of FOS is not affected by the presence of non-toxic concentrations of DON.

FOS penetration is demonstrated in phagocytic, respiratory and intestinal cells, where adequate concentrations are reached.

### FOS determination in biological matrices

There are only a few methods for FOS detection in biological matrices (Pianetti 1997; Loste *et al.*, 2002; Petsch *et al.*, 2005; Gutiérrez *et al.*, 2008; Zozaya *et al.*, 2008).

In 1980, FOS dosage by a stationary phase of octadecylsilane chemically bonded with the formation of an ion pair, or using an ion-exchange column connected to a detector anionic by flame photometry selective phosphorus atom were proposed (Chester *et al.*, 1981). Its low molecular weight, low UV absorption and lack of fluorescence, are characteristics that hinder its analysis (Yu-Ling *et al.*, 1999). For this reason, for gas chromatography analysis FOS should be derivatized, meaning that a chemical modification must be introduced into FOS to facilitate its analysis and detection (Loste *et al.*, 2002). However, the limitation of this method is that is time consuming due to derivatization steps. Other studies determine FOS by microbiological methods (Sumano *et al.*, 2007) or by capillary electrophoresis (Petsch *et al.*, 2005).

Currently, high resolution liquid chromatography coupled to a mass spectrometer (HPLC MS/MS) is the method of choice for xenobiotics determination. Its use has been described for FOS determination in serum of humans (Li, 2007), chickens (Dieguez *et al.*, 2011; Soraci *et al.*, 2011b) and piglets (Soraci *et al.*, 2011a; Pérez *et al.*, 2012b) and in broiler chicken and pig tissues (Pérez *et al.*, 2011, 2013b).

Compared with the methods mentioned above, HPLC MS/MS is the method of choice to perform these determinations due to its specificity and the lack of need for derivatization.

There are only a few methodologies for FOS detection in biological matrices. HPLC MS/MS is the method of choice for FOS determination due to its specificity and the lack of need for derivatization.

### FOS concentrations in different tissues

As mentioned above, it has been shown that FOS has a very low protein binding, and this, along with its low molecular weight and water solubility, allow good tissue diffusion.

### FOS concentrations in animal tissues for human consumption

FOS tissue residues studies have been conducted in broiler chickens and swines. Aramayona *et al*. (1997), determined, by microbiological assay, FOS residual concentrations in various tissues (kidney, liver, lung, muscle, heart, fat, gizzard) after chronic administration of the antibiotic in drinking water (150 micrograms/mL, during 5 days). At day 6 of the assay, FOS was detected in all tissues, except in muscle, in concentrations between 0.63 mg/g in fat to 13.48 mg/g in kidney. 24 hrs later, concentrations were below the detection limit of the method. Mestorino *et al*. (2011) studied the residual profile of FOS in broiler chickens after PO administration of calcium FOS (10 mg/kg b.w.) in water, for 5 days.

FOS concentrations were determined in muscle, skin/fat, liver, kidney and feathers, by microbiological assay. To determine FOS withdrawal time (WDT), Mestorino *et al*. (2011) have used the only MRL established by The Japan Food Chemical Research Foundation for bovine tissues (0.5 ppm).

In muscle, FOS concentrations were below the method detection limit (0.0625 mg/g) from the fourth day of discontinuation of FOS administration. In skin/fat concentrations of 0.337 mg/g were obtained the first day after administration, and from the second day, values were below the detection limit. The highest concentrations were found in liver, falling below the detection limit, from the fourth day after ending the treatment. In kidney, they found concentrations of 0.447 mg/g, which, on the second day, were below the detection limit. WDT for FOS in muscle and liver were determined by 1.4 WT program, being 7 and 5 days, respectively (Mestorino *et al.*, 2011). Pérez *et al*. (2011) determined FOS residual concentrations by HPLC MS/MS and WDT in muscle (pectoral, thigh and injection site), liver and kidney of broiler chickens after PO and IM administrations.

In this study, the WDTs of FOS were determined considering also the Maximum Residue Limit (MRL) defined by Japan. Concentrations of FOS in muscle, liver and kidneys were always below the MRL. In addition, after 72 h of FOS food withdrawal and IM administration, the values of the residual concentrations of the drug in tissues were below the 0.1 mg/g detection limit. FOS WDT in muscle was 1-2 days, being of 1.12 days for calcium FOS (PO assay) and 1.72 days for disodium salt (IM assay). Differences between FOS WDTs in muscle may be due to the distinct formulations and routes of administration.

The same applies to WDTs in liver and kidney, which are also longer after FOS PO calcium food consumption (1.27 vs. 0.42 days and 2.55 vs. 0.92 days, respectively). Authors conclude that a WDT of 2 days after IM administration and of 3 days after PO administration could be assigned as a precautionary principle for public health, without a significant economic impact for broiler producers.

Perez *et al*. (2013b) have also determined FOS residual concentrations and WDT in swine muscle, liver, kidney and skin/fat, after PO and IM administration. In both assays, FOS concentrations in all the matrices were below the MRL after 48 h of FOS food withdrawal and IM administration. After 72 h, the values of the residual concentrations of the drug in the analyzed tissues were below the 0.1 mg/mL detection limit of the method. FOS WDT in muscle was 2-3 days, being of 2.78 days for calcium FOS (PO assay) and 1.48 days for disodium salt (IM assay). WDTs in liver and kidney are longer for FOS after PO administration of calcium FOS in food (2.69 vs. 1.73 days and 2.95 vs. 1.38 days, respectively) (Pérez *et al.*, 2013b). No significant differences were found between the WDTs for skin-fat after the PO assay (0.9 days) and the IM assay (1.27). A WDT of 3 days for the PO administration and of 2 days for the IM administration were assigned.

FOS tissue residue studies have been conducted in broiler chickens and swines for WDT determination, after PO and IM administration of calcium and disodium FOS, respectively (Pérez *et al.*, 2011, 2013b). For both species a WDT of 3 days after PO administration and of 2 days after IM administration could be assigned as a precautionary principle for public health, without a significant economic impact for producers.

## Conclusion

FOS is a good antibiotic, with a fast effect, good tolerance and physicochemical and pharmacokinetic characteristics that allow its enteral and parenteral administration. Its pharmacokinetics has been studied in most domestic animal species. However, it is not widely used in veterinary medicine, being almost limited to intensive production of broiler chickens and pigs. The low toxicity and potential efficacy of FOS are the main factors that contribute to its use in humans and animals. This, together with the additional properties of the drug (inhibition of bacterial adhesion to epithelial cells, penetration in exopolysaccharide biofilm, immunomodulatory effect, promotion of the phagocytosis and protection against the nephrotoxicity caused by other drugs, intracellular penetration and diffusion into bacteria biophases), gives an extra value to FOS and make it a good option in the treatment of infectious diseases caused by sensitive organisms.
